# Supplementary material for: SARS‐CoV‐2 receptor ACE2 and TMPRSS2 are primarily expressed in bronchial transient secretory cells
Source: EMBO J. 2020 Apr 14;39(10):e105114. doi: 10.15252/embj.20105114 (PMC7232010; doi:10.15252/embj.20105114)

# Appendix

## Table of contents


1. **Appendix Figure legends**
2. **Appendix Figures**
    a. **Appendix Figure S1**
    b. **Appendix Figure S2**
    c. **Appendix Figure S3**
    d. **Appendix Figure S4**


## Appendix Figure legends

**Appendix Figure S1 – Exemplary characterization of an air liquid interface (ALI) culture derived from HBECs.**

Filters of an ALI culture were stained for the indicated bronchial epithelial markers and analyzed using confocal microscopy. Scale bars, 50 µm for all panels, except 'enlarged' top and side view panels, 20 µm.

**Appendix Figure S2 – Cell type identification by marker genes.**

DotPlots indicate the percent expressing cells (size) and expression level (color) for selected marker genes for each cell type in HBECs.

**Appendix Figure S3 - single-cell sequencing quality control.**

A, B Number of unique molecular identifiers (UMIs, top row) per cell for each individual sample from lung tissue (A) and HBECs (B).

C, D Number of genes per cell for each individual sample from lung tissue (A) and HBECs (B).

E Percentage of mitochondrial reads in different cell types in the lung dataset.

**Appendix Figure S4 – Expression of DPP4 in primary lung.**

A, B Expression levels (A) and percentage of positive cells (B) for *DPP4* in the lung dataset.

Appendix Fig. S1

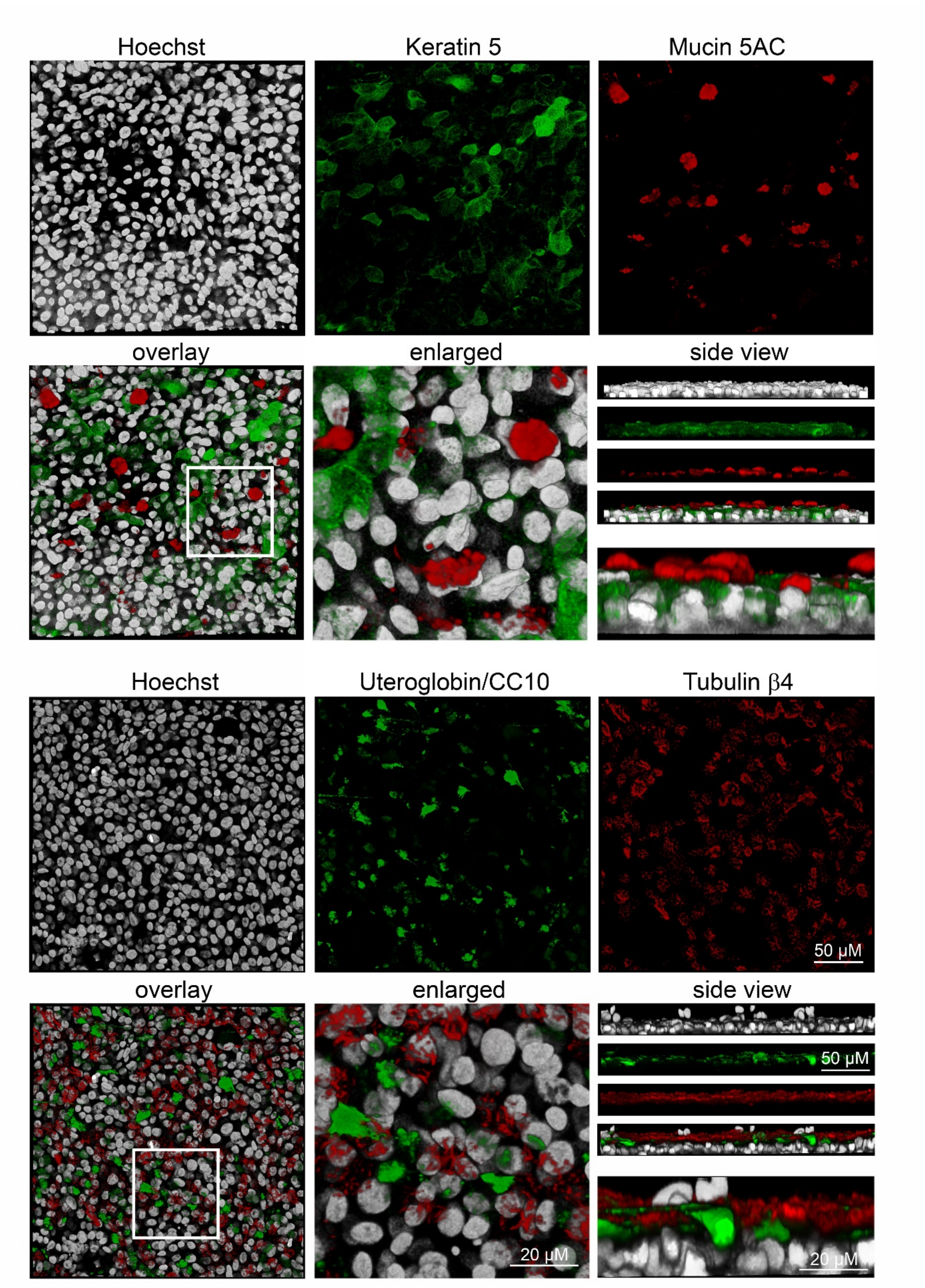

Appendix Fig. S2

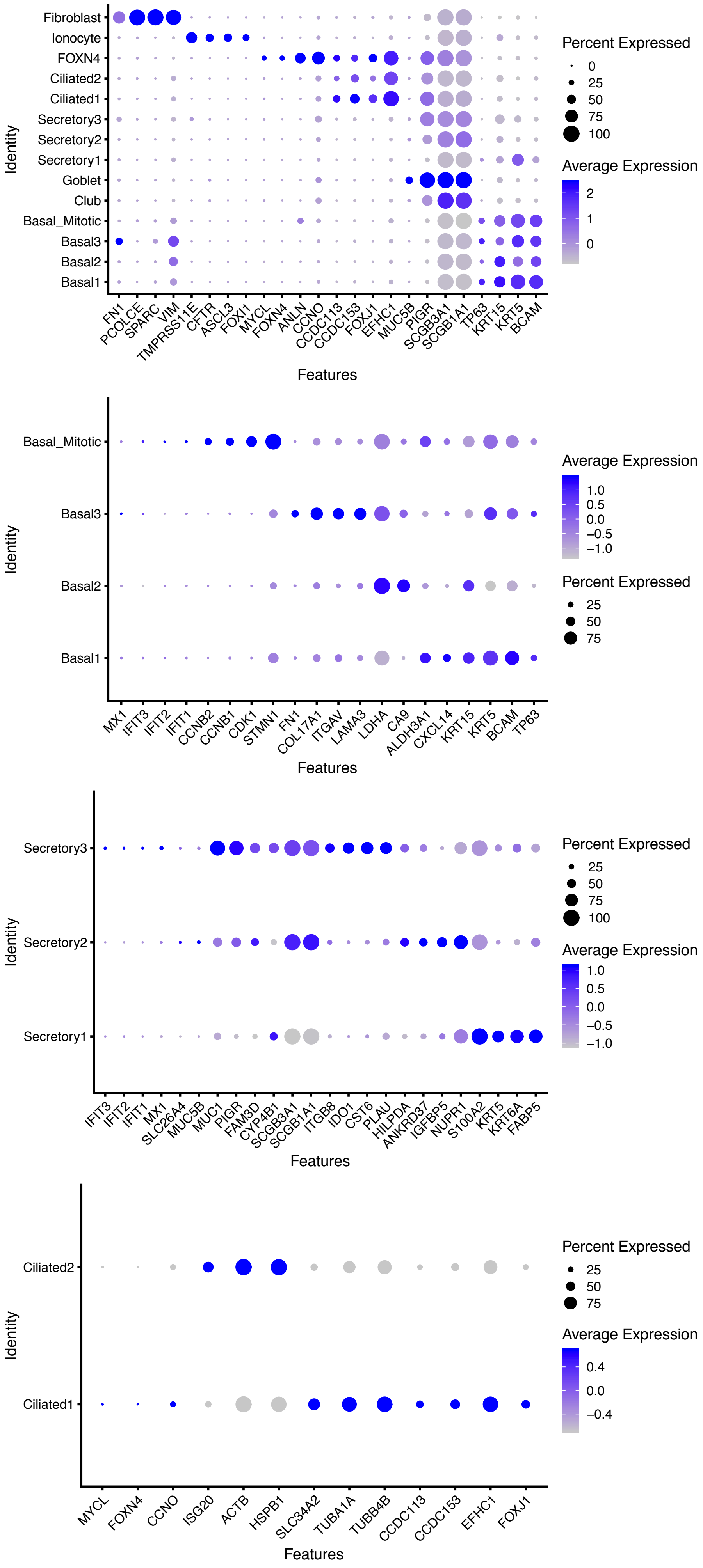

Appendix Fig. S3

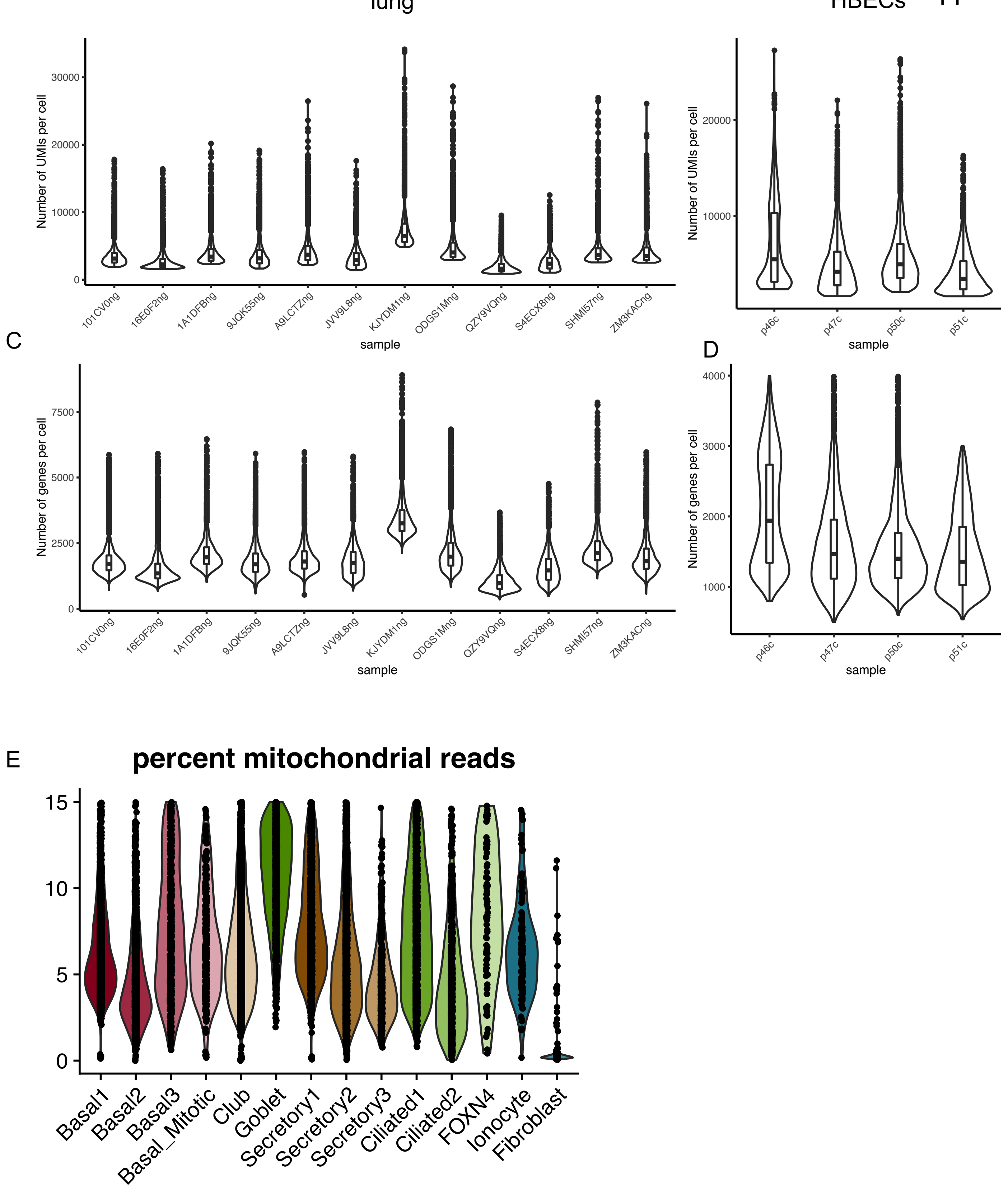

Appendix Fig. S4

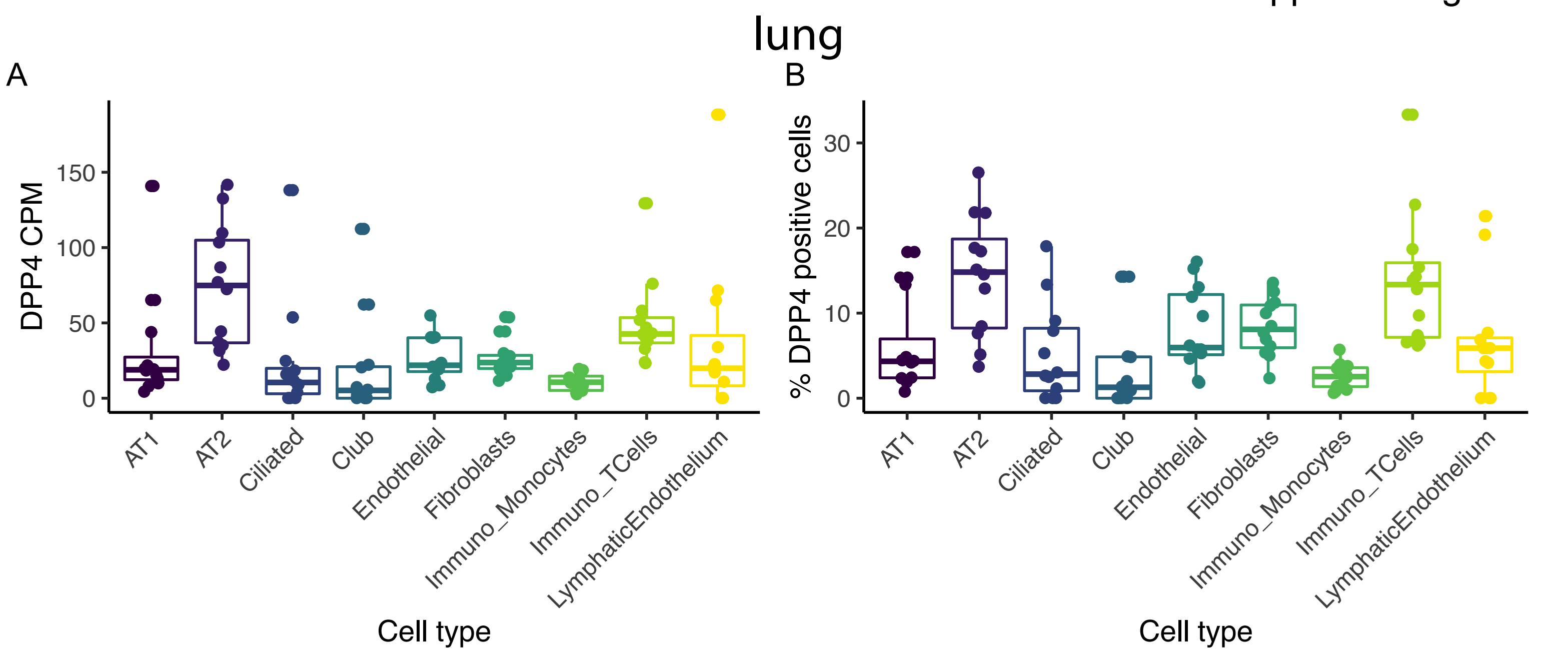

Supplement: Supplementary file 1 — Appendix [file EMBJ-39-e105114-s001.pdf]
