# Supplementary material for: SARS‐CoV‐2 receptor ACE2 and TMPRSS2 are primarily expressed in bronchial transient secretory cells
Source: EMBO J. 2020 Apr 14;39(10):e105114. doi: 10.15252/embj.20105114 (PMC7232010; doi:10.15252/embj.20105114)
Supplement: Supplementary file 2 — Expanded View Figures PDF [file EMBJ-39-e105114-s002.pdf]

Expanded View Figures

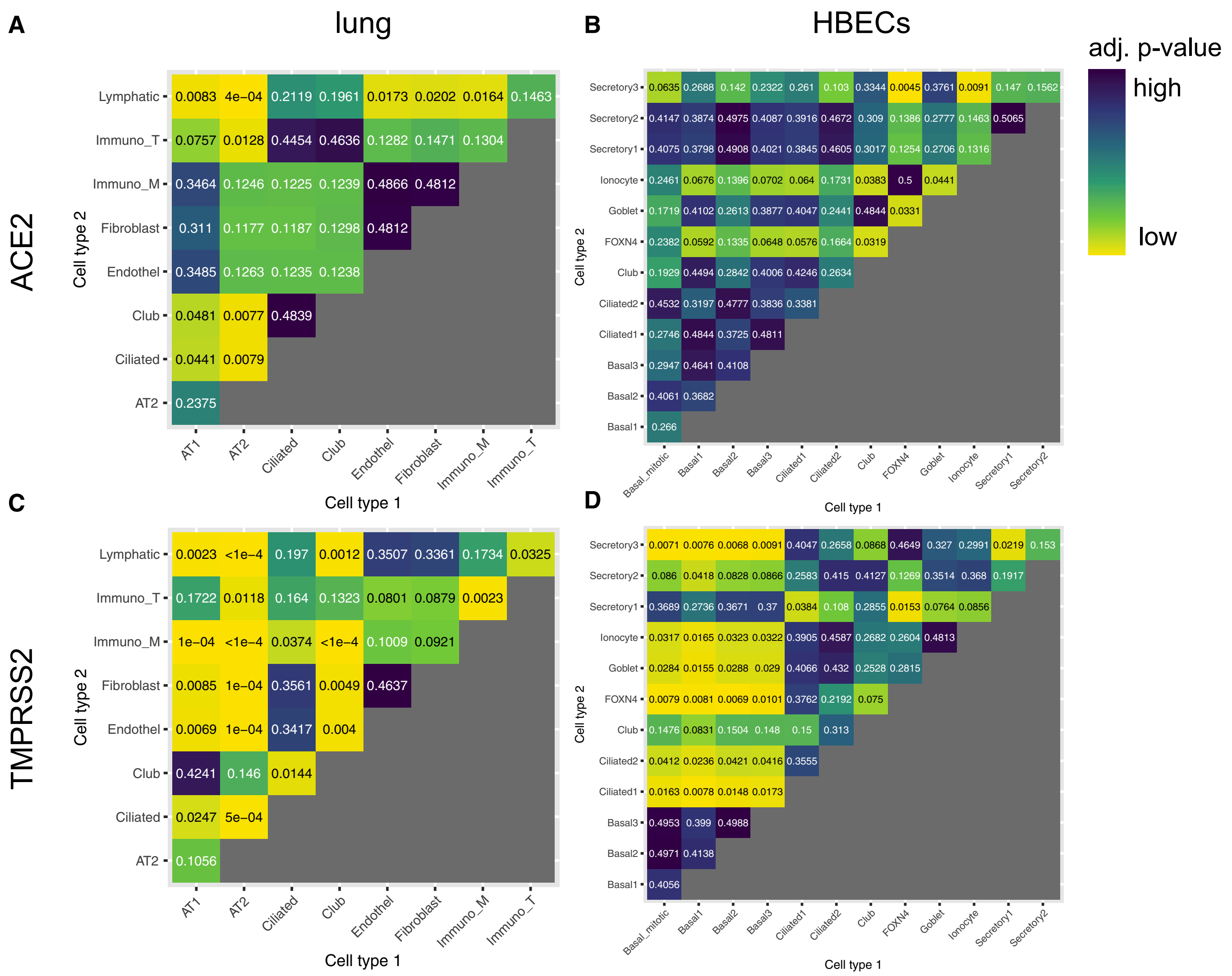

Figure EV1. Statistical analysis of *ACE2* and *TMPRSS2* expression differences.

A–D Heatmaps indicating the *P*-values of comparisons of expression levels between different cell types in the lung (A, C) and HBECs (B, D).

Data information: Values are obtained from Dunn's *post hoc* test followed by Benjamini–Hochberg correction for multiple testing.

© 2020 The Authors

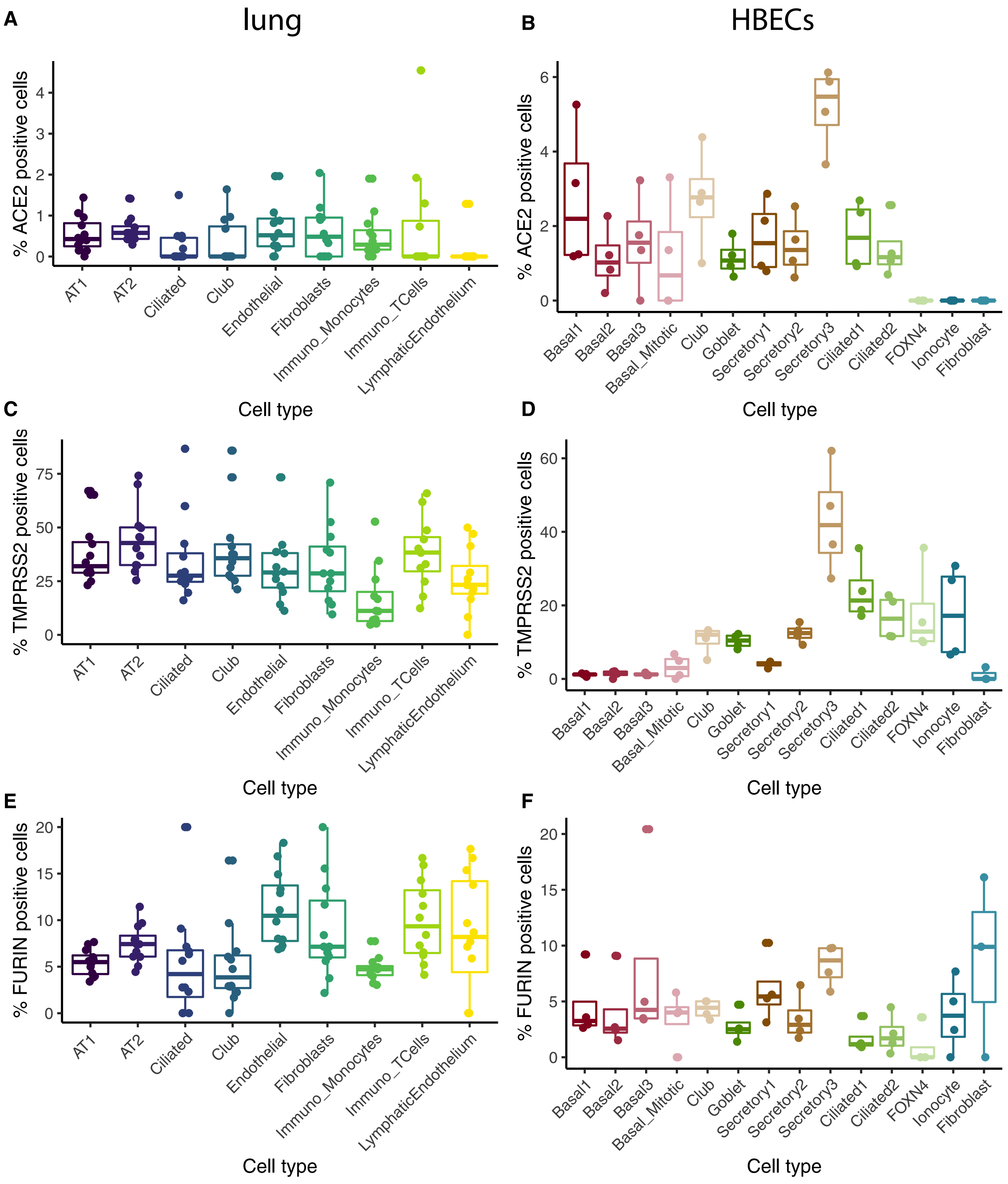

Figure EV2. Percentage of *ACE2*-, *TMPRSS2*-, and *FURIN*-positive cells.

A, B Percentage of positive cells (defined here as having at least one read of the respective gene) for *ACE2* in the lung (A) and HBECs (B).

C, D Percentage of positive cells (defined here as having at least one read of the respective gene) for *TMPRSS2* in the lung (C) and HBECs (D).

E, F Percentage of positive cells (defined here as having at least one read of the respective gene) for *FURIN* in the lung (E) and HBECs (F).

© 2020 The Authors

Figure EV3. Expression levels of *ACE2*, *TMPRSS2*, and *FURIN*.

A–C *ACE2*- (A), *TMPRSS2*- (B), and *FURIN*- (C) expressing cells in the lung plotted on top of the UMAP coordinates. Expression levels are color-coded.

D–F Dot plot indicating expression of *ACE2* (D), *TMPRSS2* (E), and *FURIN* (F) across different cell types in the lung. Percentage of expressing cells is size-coded; mean expression levels are color-coded.

G–I *ACE2*- (G), *TMPRSS2*- (H), and *FURIN*- (I) expressing HBECs plotted on top of the UMAP coordinates. Expression levels are color-coded.

J–L Dot plot indicating expression of *ACE2* (J), *TMPRSS2* (K), and *FURIN* (L) across different cell types in the bronchial epithelium. Percentage of expressing cells is size-coded; mean expression levels are color-coded.

© 2020 The Authors

© 2020 The Authors

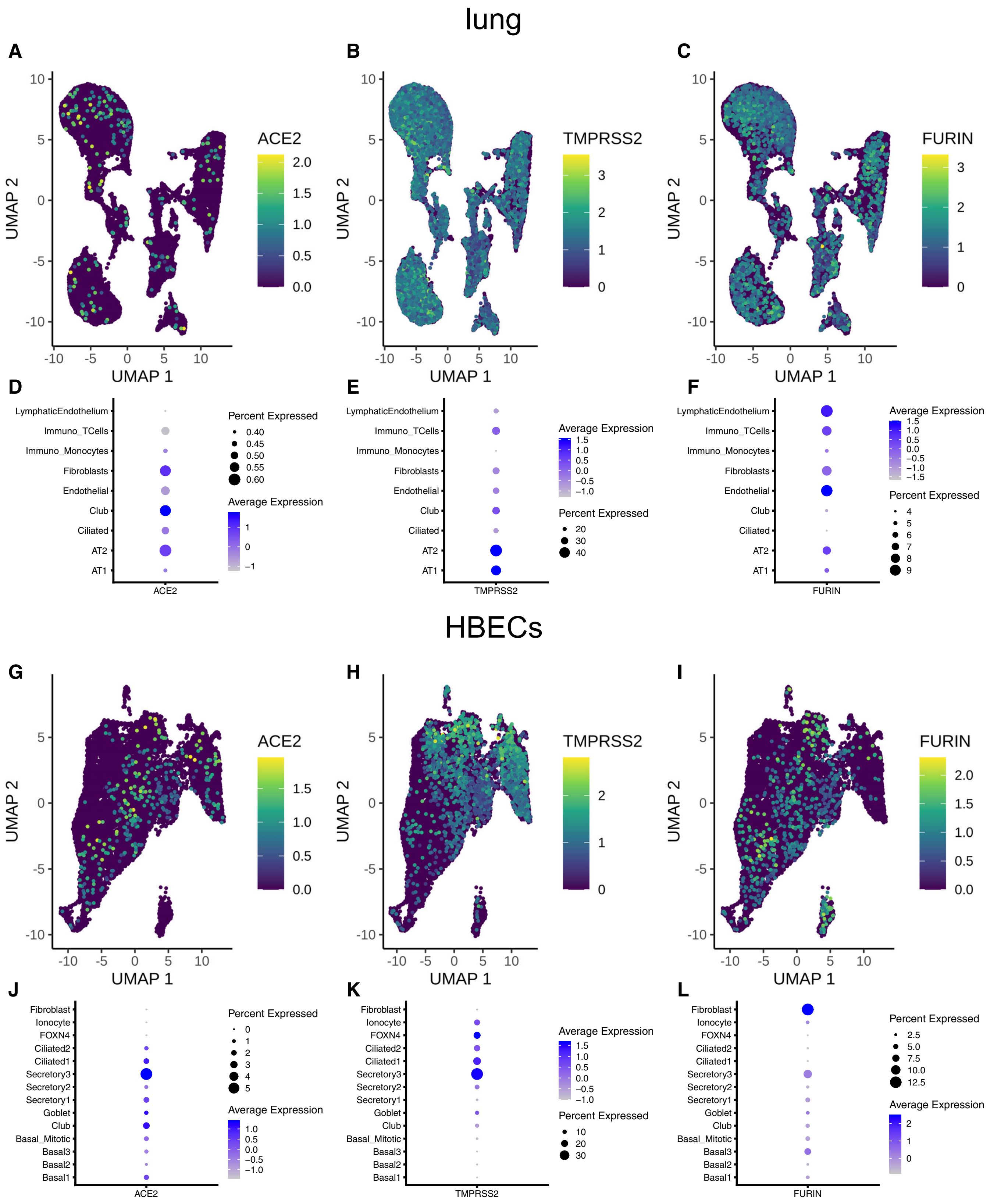
lung
A
B
C
UMAP 2
UMAP 1
ACE2
TMPRSS2
FURIN
D
E
F
LymphaticEndothelium
Immuno_TCells
Immuno_Monocytes
Fibroblasts
Endothelial
Club
Ciliated
AT2
AT1
Percent Expressed
Average Expression
HBECs
G
H
I
J
K
L
Fibroblast
Ionocyte
FOXN4
Ciliated2
Ciliated1
Secretory3
Secretory2
Secretory1
Goblet
Club
Basal_Mitotic
Basal3
Basal2
Basal1

Figure EV3.

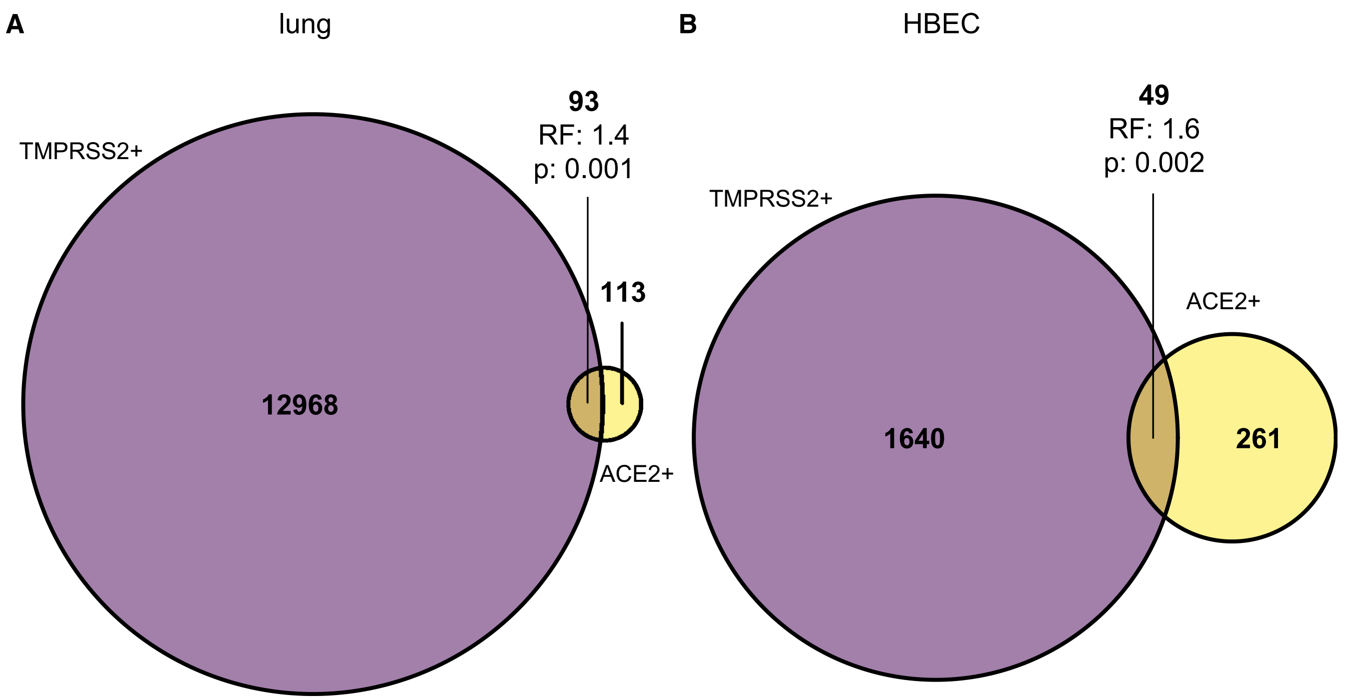

Figure EV4. Quantification of *ACE2*⁺/*TMPRSS2*⁺ double-positive cells.

A, B Overlaps and enrichment statistics for all *ACE2* and *TMPRSS2* single- and double-positive cells in the lung (A) and HBEC (B) dataset.

Data information: RF: representation factor, enrichment. P: hypergeometric tail probability.

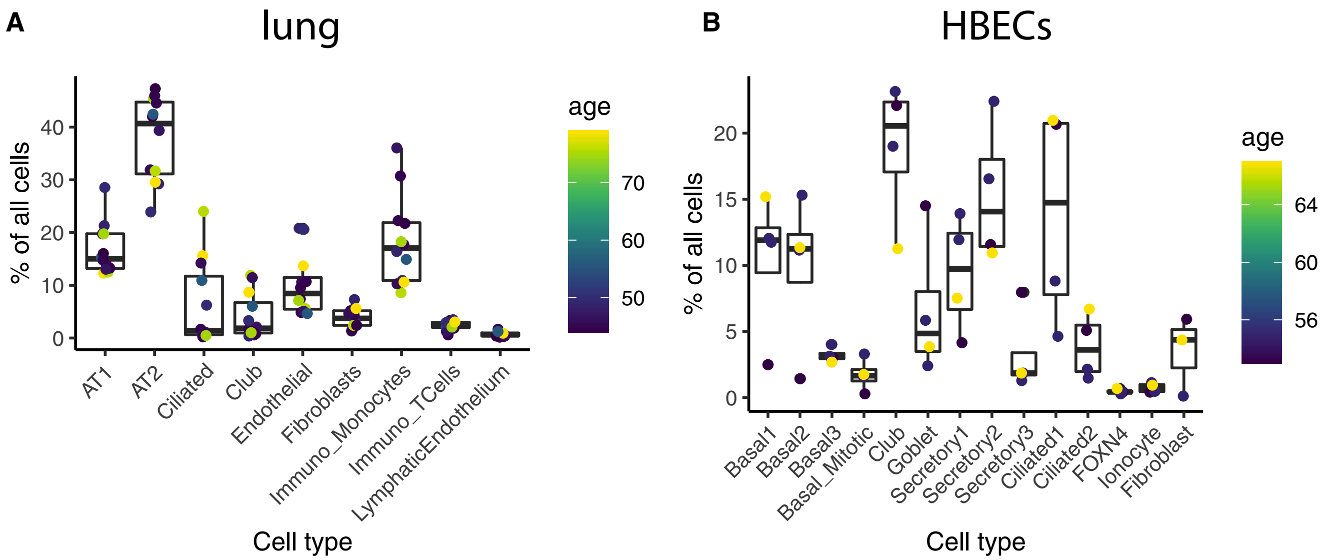

Figure EV5. Age-dependent cell type composition.

A, B Cell type composition in the lung (A) and HBEC (B) dataset. Age is color-coded.

© 2020 The Authors
